# OSATS scoring confirms ICG enhancement of performance in laparoscopic radical gastrectomy: a post-hoc analysis of a randomized controlled trial

**DOI:** 10.1097/JS9.0000000000000830

**Published:** 2023-11-07

**Authors:** Ze-Ning Huang, Qi-Chen He, Wen-Wu Qiu, Ju Wu, Chang-Yue Zheng, Guo-Sheng Lin, Ping Li, Jia-Bin Wang, Jian-Xian Lin, Jun Lu, Long-Long Cao, Mi Lin, Ru-Hong Tu, Chao-Hui Zheng, Qi-Yue Chen, Chang-Ming Huang, Jian-Wei Xie

**Affiliations:** a Department of Gastric Surgery; b Department of General Surgery, Fujian Medical University Union Hospital; c Key Laboratory of Gastrointestinal Cancer (Fujian Medical University), Ministry of Education; d Fujian Key Laboratory of Tumor Microbiology, Department of Medical Microbiology, Fujian Medical University, Fuzhou; e Department of General Surgery, Affiliated Zhongshan Hospital of Dalian University, Dalian; f Department of Gastrointestinal Surgery, the Affiliated Hospital of Putian University, Putian, People’s Republic of China

**Keywords:** gastric cancer, indocyanine green, lymph node dissection, OSATS

## Abstract

**Background:**

Indocyanine green (ICG) fluorescence imaging is effective in increasing the number of lymph node dissections during laparoscopic radical gastrectomy; however, no studies have attempted to explain this phenomenon.

**Methods:**

This study utilized the data from a previous randomized controlled trial (FUGES-012 study) investigating ICG-guided laparoscopic radical gastrectomy performed between November 2018 and July 2019. The Objective Structured Assessments of Technical Skills (OSATS) scoring system was used to grade videos from the ICG and non-ICG groups. Patients with an OSATS score greater than 29 were classified as the high-OSATS population, while those with an OSATS score less than or equal to 29 were classified as the low-OSATS population.

**Results:**

A total of 258 patients were included in the modified intention-to-treat analysis: 129 in the ICG group and 129 in the non-ICG group. The OSATS score of the ICG group was higher than that of the non-ICG group (29.6±2.6 vs. 26.6±3.6; *P*<0.001). The ICG group underwent a significantly higher mean total number of lymph node dissections than the non-ICG group (50.5±15.9 vs. 42.0±10.3; adjusted *P*<0.001). The group assigned to ICG use, better OSATS (high-OSATS) scores were observed, which correlated with greater D2 lymph node retrieval (54.1±15.0 vs. 47.2±8.7; adjusted *P*=0.039). Finally, the ICG group had a lower rate of lymph node noncompliance than that of the non-ICG group (31.8 vs. 57.4%; *P*<0.001).

**Conclusions:**

By applying the ICG fluorescence navigation technique, better OSATS scores were observed, which correlated with greater lymph node retrieval and a lower lymph node noncompliance rate, as recommended for individualized laparoscopic radical gastrectomy.

## Introduction

Highlights

- In this work, we evaluated the reasons for the increasing number of lymph node dissections during laparoscopic indocyanine green (ICG)-guided radical gastrectomy in GC patients.
- We found that the ICG fluorescent navigation technique can increase the number of lymph node dissections.
- By using the ICG fluorescence navigation technique, better Objective Structured Assessments of Technical Skills scores were observed, which correlated with greater lymph node retrieval and a lower lymph node noncompliance rate.

Gastric cancer is one of the leading cause of cancer-related deaths, with greater than 70 000 annual deaths worldwide, and the third leading cause of tumor-related deaths^1^. Currently, surgery-based comprehensive treatment remains the cornerstone of gastric cancer treatment, with D2 radical gastrectomy being the standard procedure recognized by surgeons^2^. However, due to complex perigastric lymphatic drainage and a high rate of lymph node metastasis^3,4^, more than half of the patients with progressive gastric cancer die due to disease recurrence and metastasis even after D2 radical gastrectomy. Gastrectomy has become less invasive and more precise and ensures radical tumor resection. Therefore, the quality of lymph node dissection has also become an important prognostic factor for laparoscopic radical gastrectomy^5^. As a result, thorough lymph node dissection is key for ensuring long-term tumor-free survival in patients with progressive gastric cancer^6^.

Owing to the complexity of the perigastric lymphatic system and difficulty in identifying the lymphatic adipose tissue, surgeons, especially those lacking experience, frequently omit lymph node dissection during surgery^7^. Consequently, developing a lymph node visualization system to assist surgeons in better localizing lymph nodes is a research topic of interest in this field. Indocyanine green (ICG) near-infrared (NIR) light imaging has been widely used as a new surgical navigation technique for a variety of cancer treatment procedures^8–10^. Several studies have demonstrated that the use of ICG combined with NIR can significantly increase the number of lymph node dissections in gastric cancer treatment^11–14^.

Our center conducted a randomized controlled trial (RCT). The FUGES-012 study^14^ showed that the ICG fluoroscopic navigation technique increased the number of lymph node dissections during gastrectomy and decreased the rate of lymph node noncompliance. So, we advocated for the routine use of the ICG fluoroscopic navigation technique for laparoscopic surgery in patients with gastric cancer. The mechanism by which the ICG fluoroscopic navigation technique increases the number of lymph node dissections is currently unknown. Elucidating this mechanism could provide a foundation for more precise and individualized use of the ICG fluoroscopic navigation technique. Recent studies^15,16^ have shown that improved surgical technique and reduced intraoperative errors can help surgeons in increasing the number of lymph node dissections during gastrectomy, while reducing postoperative complications and improving the long-term prognosis. Thus, we speculated that the ICG fluoroscopic navigation technique may increase the number of lymph node dissections by improving intraoperative skills. Based on cases from the FUGES-012 study, we further analyzed the role of ICG in improving the intraoperative skills for laparoscopic radical gastrectomy by introducing the Objective Structured Assessments of Technical Skills (OSATS) scoring system.

## Methods

### Study design and patient data

**Figure 1:**
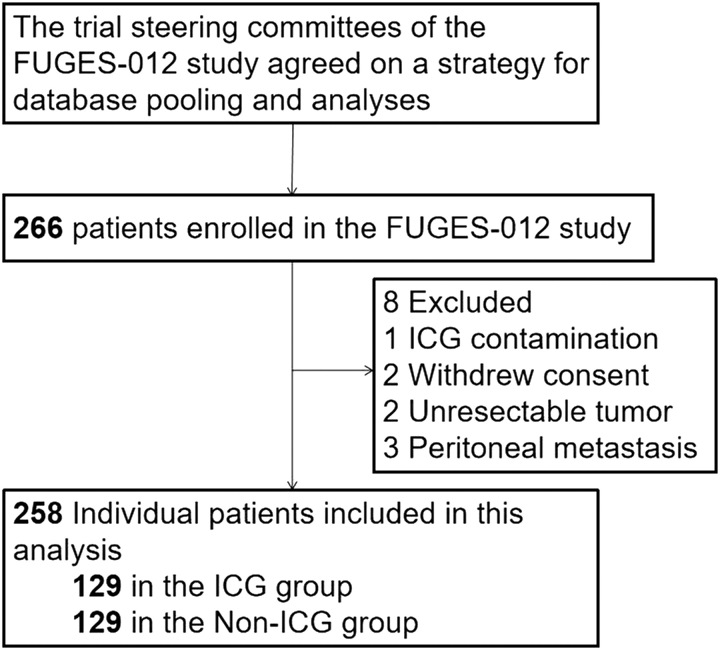
Study flowchart. FUGES, Fujian Medical University Union Hospital Gastric Surgery Study; ICG, indocyanine green.

This study analyzed data from patients with gastric cancer who participated in the FUGES-012 prospective study between November 2018 and July 2019. A total of 266 patients were included in the study. Inclusion and exclusion criteria are listed in Supplemental Table 1 (Supplemental Digital Content 3, http://links.lww.com/JS9/B285). Inclusion criteria were as follows: 1) patients aged 18–75 years; 2) primary gastric adenocarcinoma; 3) preoperative assessment indicating tumor stage cT1 to cT4a, N -/+, and M0; 4) no distant metastasis; 5) Eastern Cooperative Oncology Group (ECOG) score of 0–1, and 6) American Society of Anesthesiologists scores of grades I–III. Exclusion criteria were as follows: 1) pregnancy or lactation; 2) severe mental disorders; 3) history of upper abdominal surgery, gastrectomy, endoscopic mucosal resection, endoscopic submucosal dissection, or refusal of laparoscopic surgery; and 4) preoperative imaging showing enlarged lymph nodes with diameters greater than 3 cm. The screening process used in this study is illustrated in Figure 1. Finally, 258 patients were included in the modified intention-to-treat analysis. This study was approved by the institutional review board and is reported in line with the CONSORT^17^ and STROCSS^18^ guidelines.

### ICG fluorescence imaging

One day before the surgery, patients in the ICG group received a submucosal injection of ICG. Approximately, 1.25 mg/ml of ICG was prepared with sterile water, and the solution (0.5 ml) was injected into the submucosa in the four quadrants surrounding the primary tumor, totaling 2.5 mg of injected solution^10^. During surgery, NIR fluorescence images were obtained using the NOVADAQ fluorescent surgical system (Stryker), which allowed the surgeon to switch fluorescence modes (infrared, green fluorescence, and color-segmented fluorescence imaging) with a simple finger click.

### OSATS

The OSATS scoring system has been widely used in clinical practice since 1997 when Martin *et al*.^19^ reported the use of the OSATS global rating scale to assess the technical skills of trainee surgeons^15,16^. The OSATS global rating scale consists of seven subcategories, each with a minimum score of five points and a maximum score of 35 points^19^. The OSATS seven items scoring details are described in Supplemental Table 2 (Supplemental Digital Content 4, http://links.lww.com/JS9/B286). Intraoperative videos of the procedure for each patient were recorded, and postoperative scores for each step of the procedure were obtained. According to Fecso *et al*.^20^, a score of 29/35 was used as the cut-off value; classifying the patients into the high-OSATS population (>29) and the low-OSATS population (≤29). According to de Montbrun *et al*.^21^, the seven subcategories were defined as follows: 1) respect for tissue, 2) time and motion, 3) instrument handling, 4) knowledge of instruments, 5) use of assistants, 6) flow of operation and forward planning, and 7) knowledge of specific procedures. We used 3/5 as the cut-off value to create seven subcategories of the respective high-scoring (>3) and low-scoring (≤3) populations.

### Surgical quality control and surgical technique

All patients in this study were operated on by two experienced surgeons who had performed greater than 100 radical laparoscopic gastrectomy procedures. The gastrectomy extent was selected, and D2 lymph node dissection was performed in accordance with the Japanese Guidelines for Gastric Cancer (4th edition)^2^. Lymph node dissection sequences were performed as previously described^22^. Selective resection of the No. 10 lymph nodes was performed in cases when: 1) the primary tumor was in the upper middle part of the greater curvature of the stomach, 2) preoperative imaging suggested enlarged splenic hilar lymph nodes, or 3) the No. 10 lymph nodes fluoresced in the NIR mode^23,24^. After the completion of lymph node dissection, NIR imaging was routinely performed in the ICG group, and any residual fluorescent lymph nodes were excised. When there was a high suspicion of tumor invasion or fluorescence from the No. 14v lymph nodes, they were resected^25^.

The unedited videos of the procedure performed on each patient were reviewed and scored by two experts with surgical backgrounds. Both assessors of this study were familiar with the scoring rules of the OSATS scoring system, familiar with the process of laparoscopic radical gastrectomy, and had more than 3 years of surgical experience, and both had more than 50 cases of laparoscopic gastrectomy. During the evaluation, these experts watched the videos in real-time, observed each surgical step in slow motion, rewinding the videos if necessary, and finally filled out the form with their scores. Disagreements were resolved through consensus. The video file only contained a laparoscopic view and no additional information. The rater had no knowledge of the video source, surgeon level, or patient outcome.

### Outcome measurements

The definition of lymph node stations in the Japanese lymph node classification guidelines for gastric cancer^26^ was used to isolate lymphatic soft tissues from isolated specimens. In the ICG group, fluorescent and nonfluorescent lymph nodes were directly retrieved using the fluorescence generated by the NOVADAQ fluorescence surgery system. Stations containing fluorescent lymph nodes were defined as fluorescent stations, whereas those without fluorescent lymph nodes were defined as nonfluorescent stations.

Immediately after the fluorescence detection of lymph node stations, the specimens were sent to the pathology department. All pathological examinations were performed following established protocols described in previous studies^27^. The clinical T category (cT), clinical N category (cN), pathological T category (pT), and pathological N category (pN) of gastric cancer were determined according to the eighth edition of the American Joint Committee on Cancer (AJCC) staging manual^27^. An increase in retrieved lymph nodes (RLNs) was defined as RLNs greater than or equal to 40, as described in a multicenter study from Japan^28^. Lymph node noncompliance was defined as the absence of lymph nodes that should have been excised from more than one lymph node station.

### Statistical analysis

All data were statistically analyzed using the SPSS statistical software for Windows (version 26.0). The data were analyzed using the intention-to-process principle. Continuous variables were expressed as SD, while categorical variables were expressed as frequencies and percentages. The *χ*^2^ test or the independent *t*-test was used to evaluate the relationships between categorical and continuous data. Correlation analyses were performed by evaluating Spearman’s and Pearson’s correlation coefficients or the Eta coefficient and one-way analysis of variance, as appropriate. The mediation analysis model and directed acyclic graphs (DAGs) were used to analyze the mediating effect of the variable. All DAGs were created and analyzed using the DAGitty 3.0 software^29^. Logistic models were developed to analyze the independent predictors affecting the number of lymph node clearances and OSATS scores separately. Variables with *P*<0.1 were included in a logistic univariate analysis, and potentially relevant variables that may affect clinical outcomes were included in a multivariate model for analysis. Differences were considered statistically significant at *P*<0.05. To account for multiple comparisons, two-sided *P*-values were adjusted using the Benjamini/Hochberg (B/H) method to control the false discovery rate^30^. An association was considered to be statistically significant if its corresponding B/H-adjusted *P*-value was <0.05, corresponding to a false discovery rate of 5%.

## Results

### Baseline characteristics

Between November 2018 and July 2019, 266 patients with gastric cancer were enrolled in this RCT. After the exclusion criteria were applied, 258 patients were included in the modified intention-to-treat analysis, with 129 undergoing ICG-guided laparoscopic gastrectomy (ICG group) and 129 undergoing conventional laparoscopic gastrectomy (non-ICG group). No statistical differences were observed between the two groups in terms of age, BMI, sex, ECOG score, cT, cN, pT, pN, pathological stage (AJCC 8th), and number of metastatic lymph nodes (All *P*>0.05) (Table 1). Regarding general clinicopathological data, except for age, there were no significant differences between the high-OSATS and low-OSATS population in BMI, sex, ECOG score, tumor location, histology, lymphovascular invasion, cT, cN, pT, pN, and pathological stage (AJCC 8th) (*P*>0.05) (Supplemental Table 3, Supplemental Digital Content 5, http://links.lww.com/JS9/B287). Baseline data from the high-OSATS and low-OSATS populations in the ICG and non-ICG groups were comparable (all adjusted *P*>0.05) (Supplemental Table 4, Supplemental Digital Content 6, http://links.lww.com/JS9/B288).

**Table 1:** Demographic characteristics of patients in the ICG and non-ICG groups.

	Mean±SD / No. (%)		
Characteristic	ICG (*n*=129)	Non-ICG (*n*=129)	*P*
Age, years	57.8 (10.7)	60.1 (9.1)	0.070
BMI, kg/m^2^	23.2 (3.2)	22.8 (3.1)	0.263
Sex			0.895
Male	86 (66.7)	87 (67.4)	
Female	43 (33.3)	42 (32.6)	
ECOG PS			0.848
0	114 (88.4)	113 (87.6)	
1	15 (11.6)	16 (12.4)	
Surgical procedure			**<0.001**
Distal gastrectomy	71 (55.0)	43 (33.3)	
Total gastrectomy	58 (45.0)	86 (66.7)	
Tumor location			**<0.001**
Upper	33 (25.6)	66 (52.1)	
Middle	21 (16.3)	14 (10.9)	
Lower	75 (58.1)	49 (38.0)	
Tumor size, cm			**0.003**
≤4	93 (72.1)	70 (54.3)	
>4	36 (27.9)	59 (45.7)	
Reconstruction method			**0.002**
BI	9 (7.0)	4 (3.1)	
BII	62 (48.4)	39 (30.2)	
Roux-en-Y	58 (45.0)	86 (66.7)	
cT category			0.831
cT1	35 (27.1)	29 (22.5)	
cT2	31 (24.0)	32 (24.8)	
cT3	37 (28.7)	38 (29.5)	
cT4a	26 (20.2)	30 (23.3)	
cN category			0.452
cN0	60 (46.5)	54 (41.9)	
cN+	69 (53.5)	75 (58.1)	
pT category (AJCC 8th)			0.517
T1	42 (32.6)	39 (30.2)	
T2–T3	68 (52.7)	64 (49.6)	
T4a	19 (14.7)	26 (20.2)	
pN category (AJCC 8th)			0.565
N0	54 (41.9)	55 (42.6)	
N1	24 (18.6)	16 (12.4)	
N2	20 (15.5)	18 (14.0)	
N3a	19 (14.7)	26 (20.2)	
N3b	12 (9.3)	14 (10.8)	
Pathological ctage (AJCC 8th)			0.429
I	50 (38.8)	41 (31.8)	
II	33 (25.6)	33 (25.6)	
III	46 (35.6)	55 (42.6)	
Metastasis LNs	5.6 (11.2)	5.7 (8.9)	0.941

AJCC, American Joint Committee on Cancer; ECOG PS, Eastern Cooperative Oncology performance status; ICG, indocyanine green; LN, lymph node.

### Univariate and multivariate analyses

**Figure 2:**
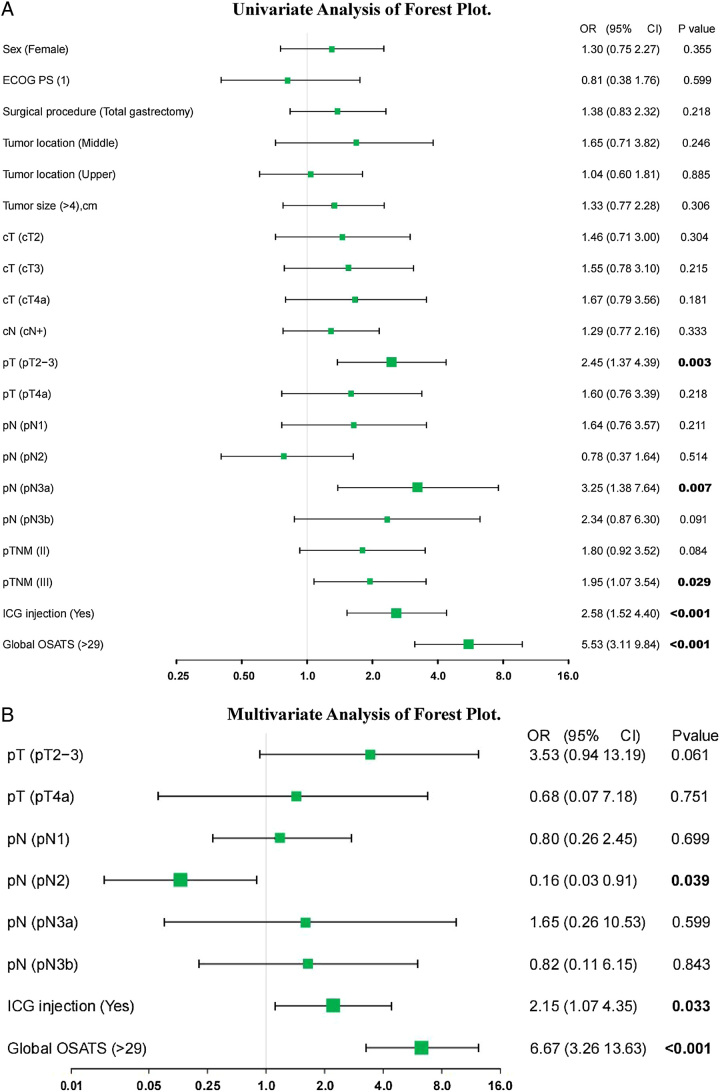
Factors affecting retrieved lymph nodes (RLNs). a, Univariate Analysis of Forest Plot; b, Multivariate Analysis of Forest Plot. ECOG, Eastern Cooperative Oncology Group; ICG, indocyanine green; OSATS, Objective Structured Assessments of Technical Skills.

Univariate and multivariate logistic regression models were used to analyze the factors affecting the number of lymph node dissections. Univariate analysis showed that T-stage, N-stage, AJCC 8th stage, OSATS score greater than 29, and ICG fluorescent navigation technique were associated with an increase in RLNs (All *P*<0.05) (Fig. 2A). In the multivariate analysis, the ICG fluorescent navigation technique [odds ratio (OR) (95% CI: 2.15 (1.07–4.35); *P*=0.033)] and OSATS score greater than 29 [OR (95% CI: 6.67 (3.26–13.63); *P*<0.001)] were independent predictive factors for RLNs greater than or equal to 40 (Fig. 2B). Variables with *P*<0.1 were included in a univariate analysis, and potentially related variables that may affect OSATS scores (sex, age, surgical procedure, reconstruction method, tumor location, tumor size, cT, cN, and ICG injection) were included in a multivariate model for analysis. After multivariate adjustment, only the ICG fluorescence navigation technique [OR (95% CI: 4.37 (2.46–7.74); *P*<0.001)] was an independent predictor of OSATS score (Table 2).

**Table 2:** Univariate and multivariate analyses for the OSATS scores in all patients.

	Univariable				Multivariable			
Clinical Parameters	OR	95% CI		*P*	OR	95% CI		*P*
Sex								
Male	Ref				Ref			
Female	1.51	0.90	2.55	0.123	1.43	0.79	2.56	0.236
Age								
<70	Ref				Ref			
≥70	0.69	0.34	1.42	0.315	0.83	0.37	1.85	0.647
Surgical procedure								
Distal gastrectomy	Ref				Ref			
Total gastrectomy	0.53	0.32	0.87	**0.012**	0.20	0.29	1.45	0.112
Reconstruction method				**0.012**				
BI	Ref				Ref			
BII	0.24	0.05	1.12	0.069	0.25	0.49	1.29	0.098
Roux-en-Y	0.14	0.03	0.66	**0.013**				
Tumor location				**0.008**				
Lower	Ref				Ref			
Middle	1.08	0.50	2.33	0.837	1.80	0.47	6.86	0.387
Upper	0.45	0.26	0.77	**0.004**	1.07	0.30	3.76	0.916
Tumor size, cm								
≤4	Ref				Ref			
>4	0.86	0.52	1.43	0.564	1.39	0.75	2.57	0.291
Histology								
Differentiated	Ref							
Undifferentiated	1.48	0.89	2.47	0.135				
cT category				0.057				
cT1	Ref				Ref			
cT2	0.80	0.40	1.62	0.539	0.87	0.39	1.96	0.732
cT3	0.40	0.20	0.80	**0.009**	0.41	0.17	1.00	0.050
cT4a	0.64	0.31	1.32	0.230	0.65	0.25	1.68	0.371
cN category								
cN0	Ref				Ref			
cN+	0.72	0.44	1.19	0.200	1.02	0.53	1.95	0.951
ICG injection								
NO	Ref				Ref			
YES	4.29	2.55	7.23	**<0.001**	4.37	2.46	7.74	**<0.001**

ICG, indocyanine green; OSATS, Objective Structured Assessments of Technical Skills.

### Surgical performance

**Figure 3:**
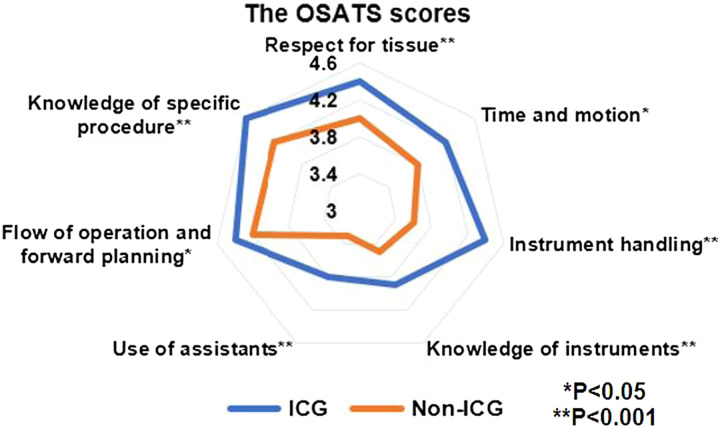
Comparison of the mean OSATS scores between the ICG and non-ICG Groups. ICG, indocyanine green; OSATS, Objective Structured Assessments of Technical Skills.

The OSATS scores of the ICG and non-ICG groups were compared using the radar-plot analysis. Compared to the non-ICG group, the ICG group associated with better scores of respect for tissue, time and motion, instrument handling, knowledge of instruments, use of assistants, flow of operation and forward planning, and knowledge of specific procedures (all *P*<0.05) (Fig. 3). Additionally, the ICG group was associated with higher OSATS scores compared to those of the non-ICG group (29.6±2.6 vs. 26.6±3.6, *P*<0.001) (Supplemental Table 5, Supplemental Digital Content 7, http://links.lww.com/JS9/B289). The proportion of patients in the high-OSATS population in the ICG group was significantly higher than that in the non-ICG group (67.2 vs. 32.8%; *P*<0.001). Similarly, there were significantly more cases of a high-OSATS score in the ICG group than in the non-ICG group in all seven subcategories (all *P*<0.05) (Table 3).

**Table 3:** Comparison of the ICG and non-ICG groups by on the basis of the seven subcategories of OSATS scores.

	No. (%)		
	ICG	Non-ICG	
Characteristic	(n=129)	(n=129)	*P*
Total OSATS			**<0.001**
≤29	41 (32.3)	86 (67.7)	
>29	88 (67.2)	43 (32.8)	
Respect for tissue			**<0.001**
1–3	13 (10.1)	42 (32.6)	
4–5	116 (89.9)	87 (67.4)	
Time and motion			**0.005**
1–3	30 (23.3)	51 (39.5)	
4–5	99 (76.7)	78 (60.5)	
Instrument handling			**<0.001**
1–3	10 (7.8)	64 (49.6)	
4–5	119 (92.2)	65 (50.4)	
Knowledge of instruments			**0.034**
1–3	57 (44.2)	74 (57.4)	
4–5	72 (55.8)	55 (42.6)	
Use of assistants			**<0.001**
1–3	50 (38.8)	92 (71.3)	
4–5	79 (61.2)	37 (28.7)	
Flow of operation and forward planning			**0.039**
1–3	14 (10.9)	26 (20.2)	
4–5	115 (89.1)	103 (79.8)	
Knowledge of specific procedure			**0.001**
1–3	9 (7.0)	27 (20.9)	
4–5	120 (93.0)	102 (79.1)	

ICG, indocyanine green; OSATS, Objective Structured Assessments of Technical Skills.

### ICG fluorescence navigation gains greater lymph node retrieval by affecting the OSATS scores

**Figure 4:**
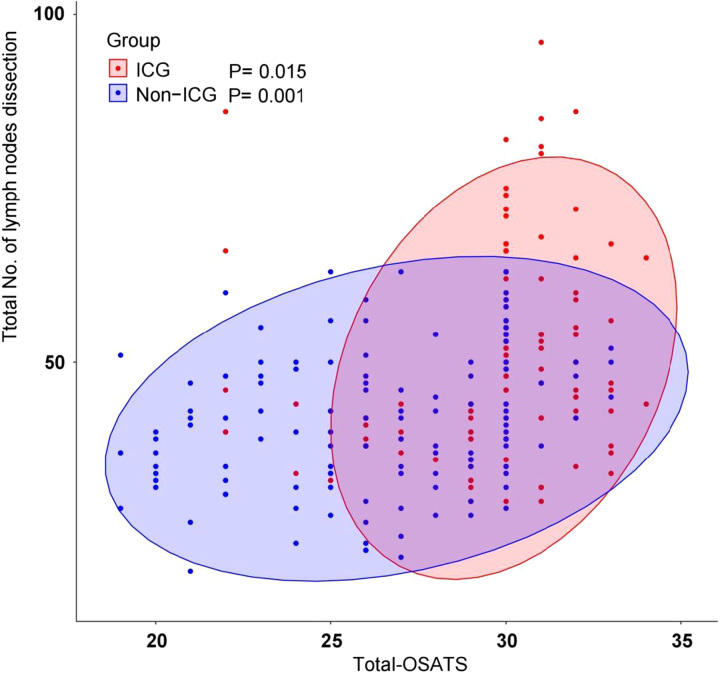
Scatter plot for total number of lymph node dissections and OSATS scores. ICG, indocyanine green; OSATS, Objective Structured Assessments of Technical Skills.

Correlation analyses showed a strong association between the ICG fluorescence navigation technique and OSATS score (F=57.71; *P*<0.001; eta-square=0.184) (Supplemental Table 6, Supplemental Digital Content 8, http://links.lww.com/JS9/B290). Similarly, there was a correlation between the total OSATS score and the mean number of total lymph node dissections (*P*<0.05) (Fig. 4). The mediation analysis model was used to analyze the difference in the number of lymph node dissections between the ICG and non-ICG groups. The results showed that the OSATS score had a significant mediating effect on the number of lymph node dissections (*P*<0.001) (Supplemental Table 7, Supplemental Digital Content 10, http://links.lww.com/JS9/B291). Furthermore, the DAG showed that a high-OSATS score mediated the ICG fluorescence navigation technology to increase the number of lymph node dissections (Supplemental Figure 1, Supplemental Digital Content 1, http://links.lww.com/JS9/B283). Further stratified analyses showed that, in all patients, the mean total number of lymph node dissections in the ICG group was significantly higher than that in the non-ICG group (50.5±15.9 vs. 42.0±10.3; adjusted *P*<0.001). In the group assigned to ICG use, better OSATS (high-OSATS) scores were observed, which correlated with greater D2 lymph node retrieval than that in the non-ICG group (54.1±15.0 vs. 47.2±8.7; adjusted *P*=0.039). Stratified analyses of the lymph node station indicated that the group assigned to ICG use, better OSATS (high-OSATS) scores were observed, which were associated with greater pylorus (9.1±6.1 vs. 6.3±2.8; adjusted *P*=0.039) and No. 5–12 regions (28.8±8.1 vs. 23.6±6.9; adjusted *P*=0.012) lymph node retrieval than that in the non-ICG group (Table 4).

**Table 4:** Comparison of retrieved lymph nodes (LNs) in the ICG and non-ICG groups between the Low-OSATS and high-OSATS populations.

	All patients				Low-OSATS (*n*=127)				High-OSATS (*n*=131)			
Group	ICG group (*n*=129)	Non-ICG group (*n*=129)			ICG group (*n*=41)	Non-ICG group (*n*=86)			ICG group (*n*=88)	Non-ICG group (*n*=43)		
Variable	Mean±SD	Mean±SD	*P*	P*	Mean±SD	Mean±SD	*P*	P*	Mean±SD	Mean±SD	*P*	P*
Total	50.5±15.9	42.0±10.3	**<0.001**	**<0.001**	40.3±9.5	39.3±9.9	0.562	0.737	55.2±16.0	47.5±8.8	**0.004**	**0.039**
D2	49.6±15.0	41.7±10.2	**<0.001**	**<0.001**	40.0±9.5	39.0± 9.8	0.567	0.737	54.1±15.0	47.2±8.7	**0.006**	**0.039**
Perigastric	31.1±12.6	27.0±9.3	**0.004**	**0.013**	23.8±8.7	25.5±9.0	0.313	0.682	34.5±12.8	30.2±9.1	**0.049**	0.142
Extraperigastric	19.4±7.2	15.0±5.8	**<0.001**	**<0.001**	16.6±4.4	13.8±5.4	**0.005**	0.130	20.7±7.8	17.4±6.0	**0.014**	0.069
Pyloric region (NO.5+6+14v)	7.9±5.6	6.0±3.2	**0.001**	**0.003**	5.3±3.2	5.9±3.4	0.387	0.682	9.1±6.1	6.3 ±2.8	**0.005**	**0.039**
Suprapancreatic region (NO.7+8+9)	12.2±5.3	9.6±4.5	**<0.001**	**<0.001**	10.6±4.1	8.9±4.0	**0.027**	0.234	13.0±5.6	11.2±4.8	0.076	0.198
NO. 1–4	23.6±11.1	21.0±8.3	**0.038**	0.062	18.4±7.3	19.6±7.8	0.435	0.682	25.9±11.7	23.9±8.6	0.316	0.533
NO. 5–12	26.6±8.0	21.0±6.8	**<0.001**	**<0.001**	21.9±5.4	19.7±6.3	0.059	0.384	28.8±8.1	23.6±6.9	**<0.001**	**0.012**
NO.1	4.1±3.3	3.2±3.3	**0.030**	0.055	3.1±2.8	2.3±2.9	0.393	0.682	4.5±3.4	4.3±3.6	0.681	0.770
NO.2	1.5±2.3	1.9±2.3	0.144	0.163	1.2±1.8	1.9±2.2	0.096	0.451	1.6±2.5	2.0±2.5	0.448	0.594
NO.3	10.2±6.8	9.6± 5.5	0.428	0.428	8.5±5.7	9.6±5.4	0.301	0.682	11.1±7.1	9.8±5.9	0.306	0.533
NO.4	7.7±5.5	6.3±4.2	**0.018**	**0.040**	5.6±3.8	5.5±3.7	0.836	0.945	8.7±5.9	7.9±4.8	0.431	0.594
NO.4sa	0.4±0.7	0.2±0.5	**0.028**	0.055	0.2±0.4	0.1±0.3	0.546	0.737	0.5±0.8	0.4±0.8	0.419	0.594
NO.4sb	1.7±1.7	1.3±1.3	**0.037**	0.062	1.2±1.3	1.1±1.2	0.693	0.858	1.9±1.8	1.6±1.4	0.328	0.533
NO.4d	5.7±4.3	4.8±3.3	0.063	0.090	4.2±3.5	4.2 ±3.0	0.985	0.985	6.4±4.6	6.0±3.4	0.613	0.724
NO.5	1.6±2.0	1.1±1.2	**0.017**	**0.040**	1.1±1.6	1.1±1.3	0.947	0.985	1.8±2.1	0.9±1.2	**0.016**	0.069
NO.6	6.0±3.8	5.0±3.1	**0.015**	**0.040**	4.2±2.7	4.8±3.2	0.339	0.682	6.8±3.9	5.3±2.8	**0.025**	0.093
NO.7	4.6±3.1	3.2±2.7	**<0.001**	**<0.001**	3.6±2.4	2.8±2.3	0.105	0.451	5.1±3.2	3.8±3.3	**0.039**	0.127
NO.8	3.9±2.7	3.3±2.6	0.096	0.119	3.3±2.1	3.0 ±2.6	0.446	0.682	4.1±2.9	4.0±2.4	0.787	0.853
NO.8a	3.7±2.5	3.3±2.6	0.17	0.184	3.3±2.1	3.0±2.6	0.446	0.682	3.9±2.7	4.0±2.4	0.964	0.964
NO.9	3.7±2.8	3.2±2.3	0.059	0.090	3.7±2.6	3.0±2.2	0.138	0.451	3.8±2.8	3.4±2.4	0.457	0.594
NO. 10	0.5±1.5	0.3±0.9	0.121	0.143	0.3±0.9	0.3±0.9	0.955	0.985	0.7±1.7	0.3±0.8	0.188	0.407
NO.11p	3.3±2.7	2.9±2.3	0.213	0.222	2.8±2.2	2.6±2.1	0.730	0.863	3.6± 2.9	3.5±2.7	0.880	0.915
NO.11d	0.4±0.8	0.3±0.6	0.084	0.113	0.4 ±0.6	0.2 ±0.6	0.156	0.451	0.4±0.8	0.3±0.7	0.562	0.696
NO.12a	2.6 ± 2.0	1.9 ± 1.7	**0.030**	0.055	2.6 ±1.7	1.8 ±1.7	**0.023**	0.234	2.6± 2.2	2.1±1.8	0.159	0.376
NO. 14v	0.3±2.2	0.0± 0.0	0.087	0.113	0.0±0.2	0.0±0.0	0.148	0.451	0.5±2.7	0.0± 0.0	0.242	0.484

ICG, indocyanine green; OSATS, OSATS, Objective Structured Assessments of Technical Skills.

P*: Adjusted *P-*values according to Benjamini/Hochberg (BH).

**Figure 5:**
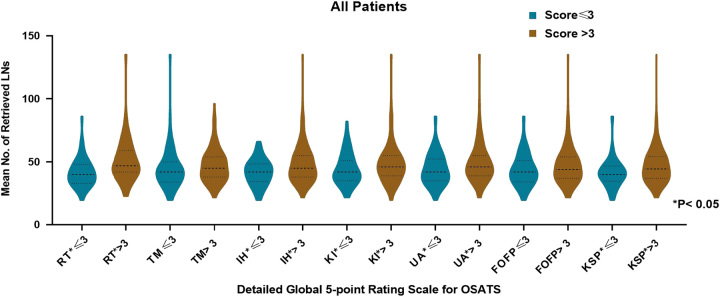
Total number of lymph node dissections stratified by the seven subcategories of OSATS scores. FOFP, flow of operation and forward planning; IH, instrument handling; KI, knowledge of instruments; KSP, knowledge of specific procedure; OSATS, Objective Structured Assessments of Technical Skills; RT, respect for tissue; TM, time and motion; UA, use of assistants.

The effect of the high-scoring (>3) and low-scoring (≤3) populations on the mean total number of lymph node dissections in the seven-item scoring rule was compared. The results showed that, in terms of respect for tissue (47.5±14.4 vs. 41.5±11.1; *P*=0.004), instrument handling (48.1±14.9 vs. 41.7±10.2; *P*=0.001), knowledge of instruments (48.3±15.3 vs. 44.3±12.3; *P*=0.021), use of assistants (49.0±15.6 vs. 44.0±12.2; *P*=0.005), and knowledge of specific procedure (47.1±14.2 vs. 41.2±11.4; *P*=0.018), the high-scoring population associated with more lymph node dissections than did the low-scoring population (all *P*<0.05) (Fig. 5).

### Stratified analysis

Further analysis of the effect of pathologic characteristics on lymph node dissection for all patients, stratified by the pT and AJCC 8th stages, showed that the high-OSATS population was associated with a higher mean total number of lymph node dissections for the same pT stage (pT1, 50.3±13.4 vs. 36.4±9.4; pT2–3, 53.8±16.1 vs. 41.5±10.2; pT4a, 54.9±11.2 vs. 39.0±7.5; all *P*<0.001). The high-OSATS population also correlated with a higher mean total number of lymph node dissections in similar AJCC 8th staging (AJCC 8th I, 50.6±12.8 vs. 36.4±8.8; AJCC 8th II, 52.6±19.8 vs. 41.5±11.6; AJCC 8th III, 54.8±12.0 vs. 41.0±8.4; all *P*<0.001) (Supplemental Table 8, Supplemental Digital Content 11, http://links.lww.com/JS9/B292). The relationship between surgical procedure and OSATS scores was analyzed and no statistical differences in scores between total gastrectomy and distal gastrectomy were observed (all *P*>0.05) (Supplemental Table 9, Supplemental Digital Content 12, http://links.lww.com/JS9/B293).

### Lymph node noncompliance

**Figure 6:**
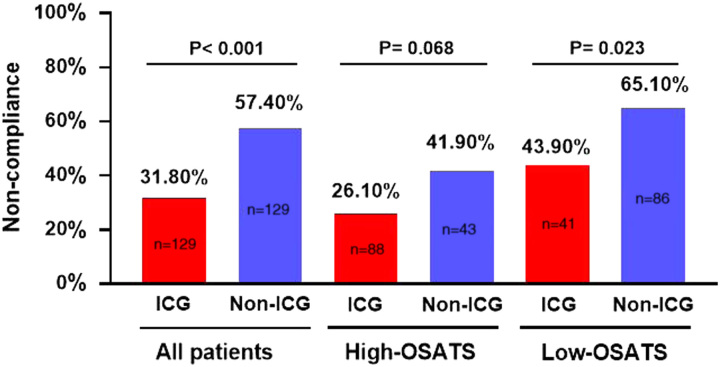
Noncompliance in lymph node dissection. ICG, indocyanine green; RT, respect for tissue; UA, use of assistants.

A comparison of lymph node noncompliance rates between the ICG and non-ICG groups showed that the ICG group had a significantly lower rate of noncompliance (31.8 vs. 57.4%; *P*<0.001). Stratified analysis revealed that, in the low-OSATS population, the lymph node noncompliance rate was significantly lower in the ICG group than that in the non-ICG group (43.9 vs. 65.1%; *P*=0.023); however, in the high-OSATS population, the lymph node noncompliance rate in the ICG and non-ICG groups was comparable (26.1 vs. 41.9%; *P*=0.068) (Fig. 6). Univariate analysis showed that the variables ʻrespect for tissueʼ, ʻinstrument handlingʼ, and ʻuse of assistantsʼ were associated with the lymph node noncompliance rate (all *P*<0.05). The multivariate analysis revealed that scores for ʻrespect for tissueʼ (RT) greater than 3 [OR (95% CI: 2.10 (1.11–4.00); *P*=0.023)] and ʻuse of assistantsʼ (UA) greater than 3 [OR (95% CI: 2.22 (1.31–3.78); *P*=0.003)] were independent predictive factors for lymph node compliance (Supplemental Table 10, Supplemental Digital Content 13, http://links.lww.com/JS9/B294). The influence of RT and UA on the lymph node noncompliance rate was analyzed and showed that, in a subgroup analysis of the low-OSATS population, in the high-RT (>3) and high-UA (>3) population, the lymph node noncompliance rate was similar in the ICG and non-ICG groups (23.5 vs. 54.5%; *P*=0.094). In the low-RT (≤3) and low-UA (≤3) population, the lymph node noncompliance rate in the ICG group was comparable to that in the non-ICG group (44.4 vs. 74.3%; *P*=0.086) (Supplemental Table 11, Supplemental Digital Content 14, http://links.lww.com/JS9/B295).

### Surgical outcomes

We found no statistically significant differences between the ICG and non-ICG groups with respect to intraoperative bleeding (51.5 vs. 54.3 ml, respectively; *P*=0.859) or operative time (196.1 vs. 190.4 min, respectively; *P*=0.333). In addition, there were no significant differences between the two groups in terms of time to first flatus, time to ambulation, time to first liquid intake, and postoperative hospital stay. The postoperative complications were also comparable between the two groups (Supplemental Table 12, Supplemental Digital Content 15, http://links.lww.com/JS9/B296). We further analyzed the influence of the OSATS score on perioperative complications and found no statistically significant differences between the high-OSATS and low-OSATS populations with respect to intraoperative bleeding (51.0 vs. 54.9 ml, respectively; *P*=0.806) or operative time (196.7 vs. 189.7 min, respectively; *P*=0.239). In terms of the postoperative recovery process, the time to first ambulation (2.1 vs. 2.5 days; *P*=0.005) and time to first semi-liquid food intake (5.3 vs. 6.2 days; *P*=0.045) was shorter in the high-OSATS population compared with that in the low-OSATS population. Overall, the prevalence of complications in the high-OSATS population was lower than that in the low-OSATS population [15 of 131 patients (11.5%) vs. 26 of 127 patients (20.5%); *P*=0.048] (Supplemental Table 13, Supplemental Digital Content 16, http://links.lww.com/JS9/B297).

## Discussion

To the best of our knowledge, this is the first study to assess the impact of ICG on surgical techniques for laparoscopic radical gastrectomy. The data analyzed in this study originated from the FUGES-012 prospective trial. Our findings showed that the group assigned to ICG use, better OSATS scores were observed, which correlated with greater lymph node retrieval and was safer and more effective than the non-ICG-guided procedure^14^. Moreover, precise tissue separation, careful and smooth instrument handling, proficiency in the use of various instruments and surgical procedures, and competent assistants may increase the number of lymph node dissections being performed. Additionally, the ICG group in the high-OSATS population correlated with more D2 lymph node dissections. Furthermore, the lymph node noncompliance rate was significantly lower in the ICG group than that in the non-ICG group. These results suggest that the ICG technique associate with increasing number of lymph node dissections and decreasing the lymph node noncompliance rate by affecting the surgical quality of laparoscopic radical gastrectomy.

Standard D2 lymph node dissection is important for the surgical treatment of gastric cancer^2,31^, and accurate staging and reduction of local recurrence^5^. However, currently, lymph node dissection is performed visually based on the operator’s experience. Due to the complex vascular anatomy and lymphatic drainage around the stomach, it is challenging to retrieve enough lymph nodes efficiently and accurately without increasing intraoperative complications, especially for inexperienced surgeons. According to previous studies, maximizing intraoperative dissection of lymph nodes would improve patient prognosis^32,33^. Elucidating how to effectively utilize existing medical resources to provide cost-effective medical programs for patients has become a new pursuit in the era of patient-centered precision surgery^34^. The ICG fluorescence navigation technology correlates with the increase of the number of lymph node dissections, albeit with some additional economic costs. As a new surgical navigation technology, ICG NIR imaging technology has shown effective results in sentinel lymph node dissection and localization of tumors, such as breast cancer and nonsmall cell lung cancer^35,36^. In our study, the ICG group correlated with a significantly higher mean total number of lymph node dissections than did the non-ICG group, which was because ICG fluoresces at a longer wavelength and penetrates deeper into the lymphatic adipose tissue^37^. The characteristics of ICG fluorescence, including its excitation light, NIR light, and tissue penetration depth of 0.5–1 cm, enable it to better identify lymph nodes in the hypertrophic adipose tissue under visible light compared to other dyes. This helps surgeons distinguish lymph nodes from other tissues, such as fat and mesangium, and organs, such as the pancreas^38,39^, and reduce the risk of overlooking lymph node dissection^40^. The progress of laparoscopic radical gastrectomy requires the tacit cooperation of the whole surgical team. ICG can improve the intraoperative performance of the whole surgical team, not just the surgical skills of the chief surgeon. Under the guidance of ICG NIR imaging, it can help the first assistant to distinguish the lymphoid tissue more clearly, so that the first assistant can maintain appropriate tissue tension in the surgical field of view and reduce the operation error of the surgeon. On the other hand, ICG can help the laparoscope holder to more easily identify perigastric anatomy to ensure a clear surgical field, that is, ICG fluorescence imaging can improve the team’s surgical coordination and operation, as well as improve the visualization level of the surgical field. Laparoscopic radical gastrectomy is a complex procedure, and surgeons often require experience with a large number of cases and extensive training to accelerate their learning. Objective assessment tools have the potential to be applied as a certification tool for surgical skill ratings; however, evidence must be provided before any scoring system can be clinically used to ensure that the scoring system adequately assesses each individual participant^41^. Cook *et al*.^42^ provided evidence for the validity of the OSATS scoring system, and previously this scale was used to score surgeons^15,16^. In this study, we use the OSATS score to assess this procedure. Lymph node dissection in the pyloric and suprapancreatic margin regions is a challenging step in laparoscopic radical gastrectomy^43^. The structures formed by the greater omentum and transverse colonic mesentery cover the anterior layer of the middle portion of the duodenum, which is adjacent to the pancreas, sometimes fused with the pancreatic peritoneum^44^, and has multiple lymphatic drainage patterns in the subpyloric and suprapancreatic margin regions^45^. Therefore, rough or careless manipulation increases the risk of complications, such as bleeding, pancreatic fistula, lymphatic fistula, and vascular injury^46–48^, which can lead to overlooking lymph node resection. Using the ICG technique not only shortens the time between omental resection and exposure of the right vein of the gastric omentum but also reduces the probability of bleeding events^49^. Our study also demonstrated that the use of the ICG technique increased the number of lymph node dissections that can be performed in the pyloric and No. 5–12 regions in cases of an OSATS score greater than 29. For surgeons, especially those lacking experience in surgery, surgical outcomes can be improved by avoiding over-clamping when using grasping forceps, which can lead to lymph node fragmentation and cause a decrease in the number of lymph nodes cleared^50^ (Supplemental Figure 2, Supplemental Digital Content 2, http://links.lww.com/JS9/B284; Supplemental Video 1, Supplemental Digital Content 17, http://links.lww.com/JS9/B298; Supplemental Video 2, Supplemental Digital Content 18, http://links.lww.com/JS9/B299). Careful manipulation and proficiency in the use of various instruments and surgical procedures can reduce the rate of intraoperative complications and increase the number of lymph node dissections^16^. Our study documented a significantly greater mean total number of lymph node dissections in the high-rated population (>3) compared with that in the low-rated population (≤3) in terms of respect for tissue, instrument handling, knowledge of instruments, use of assistants, and knowledge of specific procedures. These outcomes indicate that minimizing intraoperative errors and improving the surgical quality can increase the number of lymph node dissections.

Previous studies have also reported that the lymph node compliance rate has a significant impact on the long-term survival of patients with gastric cancer^51,52^. Therefore, we further evaluated the relationship between the OSATS scores and the lymph node noncompliance rate. The low-OSATS population demonstrated a significantly lower lymph node noncompliance rate in the ICG group relative to the non-ICG group. Furthermore, RT and UA scores greater than 3 independently predicted the lymph node compliance rate. In the high-OSATS population, the lymph node noncompliance rate in the ICG group was comparable to that in the non-ICG group owing to the satisfactory surgical skills in this population; hence, the ICG technique had little impact on the lymph node noncompliance rate in the high-OSATS population. In the low-OSATS population, the lymph node noncompliance rate in the ICG group was significantly lower than that in the non-ICG group. We believe that this was because of a fluctuation in the number of cases attributed to the small sample size in the ICG group (*n*=41/127, 32.3%). Further analysis within the low-OSATS population subgroups showed that, irrespective of high-RT (>3) or high-UA (>3) and low-RT (≤3) or low-UA (≤3) populations, there was no significant difference in the lymph node noncompliance rate between the ICG and non-ICG groups. We believe that in the low-OSATS population, although the surgical technique has not yet reached a satisfactory level, the excellent lymph node imaging results via ICG use allow the surgeon to cooperate with the assistant, which helps surgeons distinguish lymph nodes from other tissues, and reduces the risk of missed lymph node dissection. In addition, the ICG technique may correlate with reducing the lymph node noncompliance rate by affecting the surgeon’s intraoperative performance, possibly via an improved OSATS score; thus, achieving lymph node compliance is much easier. Nonetheless, further RCTs are needed to corroborate these outcomes and hypotheses.

This study had some limitations. First, although the OSATS score is the most valid assessment scale, to which most surgeons are familiar, there may be differences between assessors. The development of a standardized video assessment protocol following uniform training of assessors can help standardize assessment methods further. Second, the database for this study was derived from a prospective single-center study conducted in a Chinese hospital. Thus, the applicability of the findings to Western populations is yet to be determined. Third, to determine the oncological efficacy for long-term survival, our center is conducting survival follow-up of the patients, and preliminary results show that patients who underwent the ICG fluorescence navigation technique had significantly better 3-year OS and DFS than patients who did not undergo the ICG fluorescence navigation technique; however, the specific results require further analysis. The conclusions drawn from this study require support from other studies assessing long-term survival outcomes; the absence of which is a limitation of the present study. However, we believe that the results of this study provide useful reference values for clinical practice. ICG fluorescence navigation techniques are recommended for laparoscopic surgery in patients with gastric cancer.

## Conclusions

By using the ICG fluorescence navigation technique, better OSATS scores were observed, which correlated with greater lymph node retrieval and a lower lymph node noncompliance rate. Therefore, the ICG fluorescent navigation technique should be applied in laparoscopic radical gastrectomy to improve the quality of lymph node dissection.

## Ethical approval

The study was approved by the Institutional Review Board of Fujian Medical University Union Hospital.

## Consent

Not applicable.

## Sources of funding

This work was supported by scientific and technological innovation joint capital projects of Fujian province (2018Y9041) and Fujian province medical ʻCreate Double Highʼ construction funding [Fujian Health Medical Policy (2021) No. 76].

## Author contribution

Z.-N.H., Q.-C.H., W.-W.Q., and J.-W.X.: conception and design; Z.-N.H., Q.-C.H., W.-W.Q., C.-H.Z., P.L., and C.-M.H.: data analysis and interpretation; Z.-N.H., Q.-C.H., and W.-W.Q.: manuscript writing. All authors contributed in provision of study materials or patients, collection and assembly of data, final approval of manuscript, and accountable for all aspects of the work.

## Conflicts of interest disclosure

There are no conflicts of interest or financial ties to disclose from any authors.

## Research registration unique identifying number (UIN)

1. Name of the registry: Clinical Trials.gov.
2. Unique identifying number or registration ID: NCT03050879.
3. Hyperlink to your specific registration (must be publicly accessible and will be checked): https://clinicaltrials.gov/ct2/show/NCT03050879?term=NCT03050879&draw=2&rank=1.

## Guarantor

Qi-Chen He and Jian-wei Xie.

## Provenance and peer review

Not commissioned, externally peer-reviewed.

## Availability of data and materials

The datasets used and/or analyzed during the current study are available from the corresponding author on reasonable request.
